# Post-Extubation Stridor Complicating COVID-19-Associated Acute Respiratory Distress Syndrome: A Case Series

**DOI:** 10.7759/cureus.10492

**Published:** 2020-09-16

**Authors:** Joseph V Moran, Sara A Godil, Breanna Goldner, Kareem Godil, Jonaid Aslam

**Affiliations:** 1 Internal Medicine, Lehigh Valley Health Network, Allentown, USA; 2 Pulmonary and Critical Care Medicine, Lehigh Valley Health Network, Allentown, USA

**Keywords:** airway extubation, extubation failure, covid-19, sars-cov-2, acute respiratory distress syndrome [ards]

## Abstract

Post-extubation stridor is a known complication of mechanical ventilation that affects a substantial number of all critical care patients and leads to increased morbidity and mortality. Common risk factors for the development of post-extubation stridor include female gender, older age, and prolonged length of mechanical ventilation. There may be an increased incidence of post-extubation stridor in patients who require mechanical ventilation to manage the respiratory complications of COVID-19. In this case series, we analyzed nine patients from across our institution who were intubated to manage acute respiratory distress syndrome (ARDS) secondary to COVID-19 and subsequently developed post-extubation stridor. The patients were predominantly females with prolonged intubations and multiple days of prone ventilation. While the patients in this case series possessed some of the well-described risk factors for post-extubation stridor, there may be risk factors specific to severe acute respiratory syndrome coronavirus 2 (SARS-CoV-2) infection that make these patients more susceptible to the complication. The cuff leak test was performed on the majority of patients in the case series and did not successfully predict successful extubation in this population. Our analysis suggests that prophylactic corticosteroids given in the 24-48 hours prior to elective extubation in female COVID-19 patients who were intubated for more than six days with consecutive days of intermittent prone ventilation may be helpful in reducing the incidence of post-extubation stridor in this population. Overall, this case series elucidates the need for exceptionally close monitoring of COVID-19 patients upon extubation for the development of stridor.

## Introduction

Post-extubation stridor affects approximately 10% of all critically ill patients and is associated with re-intubation, prolonged duration of mechanical ventilation, and increased need for tracheostomy placement [1]. Patients who require mechanical ventilation to manage the complications of coronavirus disease 2019 (COVID-19), caused by severe acute respiratory syndrome coronavirus 2 (SARS-CoV-2), appear to have a substantial risk of their clinical course being complicated by post-extubation stridor [2]. In this case series, we present nine patients who required mechanical ventilation for acute respiratory distress syndrome (ARDS) secondary to COVID-19 and later developed post-extubation stridor. We will discuss the most common risk factors present in these patients, potential mechanisms for which SARS-CoV-2 makes patients more susceptible to post-extubation stridor, and potential treatment and prevention options.

## Case presentation

We identified nine patients from our institution who developed post-extubation stridor after intubation for ARDS secondary to COVID-19. During the interval that these patients were extubated, a total of 20 COVID-19 patients were extubated at our facility revealing an incidence of 45%. The patients were predominantly female with prolonged intubations and multiple days of prone ventilation. Four patients received systemic steroids for an indication other than laryngeal edema during their initial intubation with the remainder not receiving systemic steroids until they developed post-extubation stridor. Prior to their initial extubation, seven patients were noted to have a cuff leak. Six patients were initially extubated to either high flow nasal cannula or noninvasive ventilation with one of these patients avoiding re-intubation. In total, eight patients required re-intubation with four ultimately requiring tracheostomy (Table 1).

**Table 1:** Clinical Characteristics of the Patients BiPAP, bilevel positive airway pressure ventilation

Characteristics	Patients (N=9)	Percentage (%)
Mean age (range), years	65±8 (53-79)	N/A
Female sex	7	78
Body mass index	30.8±9.8	N/A
Comorbidities		
Asthma	1	11
Hypertension	5	55
Diabetes mellitus	2	22
Chronic kidney disease	1	11
Hospital day of intubation	3.5±2	N/A
Endotracheal tube size (range), mm	7±0.5	N/A
Duration of first intubation, days	13±3.7	N/A
Patient proned	9	100
Patient proned for >2 consecutive days	8	89
Paralytic used	9	100
Duration of paralytic, days	2±0.9	N/A
Cuff leak prior to extubation		
Yes	7	78
No	0	0
Not documented	2	22
Corticosteroid use prior to initial extubation	4	44
Racemic epinephrine post initial extubation	7	78
Administered once	3	43
Administered twice	4	57
Re-intubation	8	89
Duration of second intubation, days	5±3	N/A
Post initial extubation ventilation		
BiPAP	2	22
Optiflow™	4	44
Nasal cannula	3	33
Tracheostomy	4	44
Mortality		
14 days	0	0
28 days	1	11

## Discussion

The patients presented in this case series possessed some of the well-described risk factors for the development of post-extubation stridor, specifically prolonged mechanical ventilation (9/9 were >8 days), obesity (6/9 had BMI >26.5), and female gender (7/9) [3]. There was concern that due to pre-existing obesity in many of the COVID-19 patients, along with the use of enhanced personal protective equipment (PPE) for anesthesia teams that there would be an increased incidence of traumatic intubations, another common risk factor for post-extubation stridor [2]. However, it is noteworthy that no traumatic intubations were reported among these patients by the intubating team at our institution.

Another interesting correlation was that all but one of the patients in this case series required consecutive days of intermittent proning. In the 2013 Proning Severe ARDS Patients (PROSEVA) trial, which randomized 237 ARDS patients to intermittent proning, there was not a significant increase in post-extubation stridor reported in complications [4]. It is not yet clear if this is also true for COVID-19 patients who undergo proning and further analysis is needed to determine if proning is also a risk factor for post-extubation stridor in this population.

There may be risk factors specific to COVID-19 that increase the prevalence of post-extubation stridor in this population. During the interval that these nine patients were extubated, a total of 20 COVID-19 patients were extubated across our institution. This is notable considering that post-extubation stridor affects less than 10% of all critically ill patients [1]. Clinicians in smaller case series have noted considerable laryngeal edema and inflammation present upon intubation [2]. While not yet described in SARS-CoV-2, many of the other members of the coronavirus family have been associated with the development of laryngitis [5]. A study analyzing the characteristics of a breakout of human coronavirus OC43 amongst 501 patients revealed that 3.3% developed laryngitis as a clinical symptom. If laryngitis occurs in a percentage of patients with COVID-19, this could help explain why these patients are at a seemingly higher risk of post-extubation stridor.

Seven of the patients were noted to have a qualitative cuff leak prior to extubation, with three of them having a documented quantitative cuff leak of 30%, 24%, and 70%, respectively. Prior data correlate a successful extubation with a quantitative cuff leak of greater than 10%-15% [6]. However, at least one prior study suggested that the positive predictive value and sensitivity of the cuff leak test are quite low, making it a poor predictor of the presence of laryngeal edema in intubated patients. Since this assessment may not provide an accurate evaluation of the presence of laryngeal edema or predict the occurrence of post-extubation stridor, some clinicians may prefer to avoid the cuff leak test in COVID-19 patients altogether to reduce the risk of aerosolization of the virus. 

With the identification of potential risk factors, a focus can shift to proposed methods of prevention. While post-extubation stridor is often treated with systemic steroids and racemic epinephrine, the utility of prophylactic treatment prior to extubation with either agent has not been formally established. A meta-analysis on the use of prophylactic corticosteroid administration to decrease post-extubation stridor concluded that the administration of corticosteroids before elective extubation was associated with a significant reduction in the incidence of post-extubation stridor [7]. Only three of these patients were in the midst of a steroid course while being extubated, and amongst this group is the one patient who avoided re-intubation. Therefore, it may be beneficial to prophylactically administer corticosteroids one to two days prior to an elective extubation for the dedicated purpose of reducing the risk of post-extubation stridor in select patients with COVID-19, particularly those with known risk factors.

Limitations of our study include the fact that our observations of stridor are mostly based on subjective assessments rather than objective findings of airway edema seen on direct visual inspection or imaging. Additionally, there are a relatively small number of patients in our case series. 

## Conclusions

Post-extubation stridor in COVID-19 patients is an important clinical entity that has not yet been described in detail in literature and warrants further study. This case series elucidates the need for close monitoring of COVID-19 patients upon extubation for the development of stridor. Contributing to an increased prevalence of this complication may be risk factors specific to COVID-19, with viral-induced laryngeal inflammation being a distinct possibility. The presence of a cuff leak did not predict successful extubation and may be deferred to minimize aerosolization of the virus. To mitigate the risk of post-extubation stridor, the use of prophylactic corticosteroids in the 24 to 48 hours leading up to extubation in females with a ventilator time greater than six days and who have required consecutive days of intermittent proning may be helpful. With mortality rates of COVID-19 patients who require mechanical ventilation being exceptionally high, the reduction of any complication is paramount to the success of treating patients and reducing morbidity and mortality. 
